# Paternalism, autonomy and reciprocity: ethical perspectives in encounters with patients in psychiatric in-patient care

**DOI:** 10.1186/1472-6939-14-49

**Published:** 2013-12-06

**Authors:** Veikko Pelto-Piri, Karin Engström, Ingemar Engström

**Affiliations:** 1 Psychiatric Research Centre, Örebro County Council, Box 1613, SE 701 16, Örebro, Sweden; 2 School of Health and Medical Sciences, Örebro University, SE 701 82, Örebro, Sweden; 3 Department of Child and Youth Studies, Stockholm University, Frescati Hagväg 24, SE 106 91, Stockholm, Sweden

**Keywords:** Staff, Psychiatric care, Paternalism, Autonomy, Reciprocity, Qualitative theory-guided content analysis, Diary method, Ethical considerations, Ethical issues

## Abstract

**Background:**

Psychiatric staff members have the power to decide the options that frame encounters with patients. Intentional as well as unintentional framing can have a crucial impact on patients’ opportunities to be heard and participate in the process. We identified three dominant ethical perspectives in the normative medical ethics literature concerning how doctors and other staff members should frame interactions in relation to patients; paternalism, autonomy and reciprocity. The aim of this study was to describe and analyse statements describing real work situations and ethical reflections made by staff members in relation to three central perspectives in medical ethics; paternalism, autonomy and reciprocity.

**Methods:**

All staff members involved with patients in seven adult psychiatric and six child and adolescent psychiatric clinics were given the opportunity to freely describe ethical considerations in their work by keeping an ethical diary over the course of one week and 173 persons handed in their diaries. Qualitative theory-guided content analysis was used to provide a description of staff encounters with patients and in what way these encounters were consistent with, or contrary to, the three perspectives.

**Results:**

The majority of the statements could be attributed to the perspective of paternalism and several to autonomy. Only a few statements could be attributed to reciprocity, most of which concerned staff members acting contrary to the perspective. The result is presented as three perspectives containing eight values.

•*Paternalism*; 1) promoting and restoring the health of the patient, 2) providing good care and 3) assuming responsibility.

•*Autonomy*; 1) respecting the patient’s right to self-determination and information, 2) respecting the patient’s integrity and 3) protecting human rights.

•*Reciprocity*; 1) involving patients in the planning and implementation of their care and 2) building trust between staff and patients.

**Conclusions:**

Paternalism clearly appeared to be the dominant perspective among the participants, but there was also awareness of patients’ right to autonomy. Despite a normative trend towards reciprocity in psychiatry throughout the Western world*,* identifying it proved difficult in this study*.* This should be borne in mind by clinics when considering the need for ethical education, training and supervision.

## Background

The set of values that are held by psychiatric staff can make a difference in patient encounters. These values are not always obvious in everyday work since we only tend to notice them when they differ or are conflicting [1,2]. In such situations values will become apparent and standard ways of interacting with patients may be challenged. Since the relation between the patient and the caregiver is asymmetric, staff members have the power to decide the options that frame encounters with patients. Intentional as well as unintentional framing can have a crucial impact on patients’ opportunities to be heard and participate in the process [3].

Our theoretical point of departure is based on ethical perspectives that can be found in different declarations concerning professional ethics in medicine in general and psychiatry in particular. We have found three ethical perspectives that form the basis for the encounter with the patient; paternalism, autonomy and reciprocity [4]. These perspectives may be understood in relation to the historical development of normative medical ethics, but there is also a considerable overlapping between the three perspectives, which means that they may be found concurrently in our time. An important difference between the three perspectives is, the ideal about who should have the right to plan and make decisions about care and treatment.

The oldest of these perspectives is paternalism, which originated in The Hippocratic Oath [5] (Table 1). In this perspective the physician plans and decides about the treatment and care, which usually is named a paternalistic model of decision [6]. The main reason for this was that the knowledge about medical disorders came from the physician and the patients were expected to comply with these expert decisions. During World War II some physicians used their position taking measures that clearly violated the patient’s dignity and human rights. The discussion on medical ethics after the war therefore aimed to strengthen the patient’s rights. The concept of autonomy came into focus as a trump value and has been so since then within medical ethics [7]. A central document based on this perspective is The World Medical Association’s International Code of Medical Ethics [8]. Thus, the second perspective on medical encounters that can be found in declarations on professional ethics is autonomy where the decisions about treatment is primarily taken by the informed patient [6]. The third perspective is reciprocity*;* highlighted in The Madrid [9] and The Kobe Declarations [10] issued by the World Psychiatric Association. A core value in this perspective is mutual respect and co-operation which means that staff always should work in partnership with the patients, their families and other important actors, and give them a real opportunity to participate in mental health care planning and treatment. In this perspective the ideal is a shared decision-making [6].

**Table 1:** **An overview of the three ethical perspectives**[5,6,8-11]

**Perspective**	**Core normative document(s)**	**Core values highlighted**	**Decision made by (decision-model)**
Paternalism	The Hippocratic Oath	Beneficence Nonmaleficence	Professionals (Paternalistic)
Autonomy	The International Code of Medical Ethics	Autonomy	The informed patient (Informed)
Reciprocity	The Madrid declaration	Participation	The patient and staff, in association with other stakeholders (Shared)
	The Kobe declaration	Justice	

In paternalism, staff should only use their knowledge and skills for the benefit of the patient, never do harm (the “primum non nocere” principle) and always act only in the patient’s best interest. These principles are still at the heart of contemporary medical ethics*,* where beneficence and nonmaleficence are core values [11]. The Hippocratic Oath also states that health care professionals are bound by confidentiality, but no other patient rights are specified. In contrast, there is a long description of the importance of being loyal to colleagues. Paternalism emphasizes that staff members must ensure the patient’s best interest in everyday care and treatment, but that decisions are to be taken by the professionals only [6,11]. The patient is expected to comply with decisions despite the fact that the professionals may not have fully taken into account her/his specific needs and preferences [12]. This power imbalance between caregiver and patient has been problematized by political, especially feminist, writers e.g. [13] and by researchers in sociology, philosophy and psychiatry e.g. [14,15].

The idea of autonomy was clearly expressed in the first version of The International Code of Medical ethics [8] adopted in 1949. The code states that the physician is obliged to respect a competent and well-informed patient’s right to accept or refuse treatment. A key idea in this perspective is that the competent and well-informed patient has the right to make a decision, even if this is contrary to her/his best interest from a professional perspective [6,12]. Exceptions from this rule can be found in psychiatry where coercive care is possible, but only if “the patient cannot form a judgement as to what is in his or her own best interest and without which treatment serious impairment is likely to occur to the patient or others” as stated in the Hawaii declaration §5 [16]. Hence, autonomy is one of the core values of medical ethics [11] and has become increasingly dominant, especially in the Western world. Many philosophers and psychiatrists have criticized its importance since the autonomy of patients in need of psychiatric care is often diminished by the mental disorder [17]. If medical professionals place too much emphasis on the psychiatric patient’s autonomy, it may result in severe consequences, according to this view. At worst, the patient may even die because of respect for her/his wish to decline care [17,18]. When the patient’s autonomy is impaired, a long-term plan should be put in place for how to restore the patient’s capacities to enable her/him to become a partner in decision-making [17,19].

In psychiatry, reciprocity in the relationship is emphasized in the Madrid [9] and Kobe declarations [10]. The Madrid declaration states that the relationship must be based on mutual trust and respect while the Kobe declaration focuses on the family perspective. The patient and her/his family are expected to participate as full partners in the delivery of mental health care. The notion of being a full partner indicates that participation is a core value. The declarations also state that psychiatric professionals should act at community level to support patients to obtain the health care, education, employment and housing they need. This can also be seen as a plea for justice, which is considered to be a core value in medical ethics [11].

When the perspectives are presented schematically, as in Table 1, they appear to be conflicting. On the other hand, they may be seen as complementary, representing three different and valuable contributions to the ethics of psychiatry. While there is a great deal of normative literature about these perspectives, few studies are available about if and how these ethical perspectives can be found in everyday work in psychiatry. The aim of this study was to describe and analyse statements describing real work situations and ethical reflections made by staff members in ethical diaries in relation to three central perspectives in medical ethics; paternalism, autonomy and reciprocity.

## Methods

### Setting and participants

The study was conducted in seven psychiatric clinics for adults (four that offered general psychiatric care, two forensic psychiatric care and one addiction care) and six child and adolescent psychiatric clinics. All clinics had the surrounding region as their respective catchment area. The clinics chosen can be considered to be regular Swedish psychiatric clinics with heterogeneous patient populations managed by six county councils in central Sweden. Staff members on the wards who worked directly with the patients were invited to participate in the study, regardless of occupational status. We only obtained background information such as professional category, education and age, from the adult psychiatric wards. Staff members in child and adolescent psychiatry were, however, guaranteed anonymity, as there are relatively few employees in each county. Approximately half of the staff members were mental health care assistants. Registered nurses constituted the majority of the other half, while the remainder comprised various professional categories such as psychiatrists, psychologists, social workers and teachers.

### Design and procedure

The participants were asked to keep an ethical diary for a period of one week. The diary had eight pages; the first contained instructions and the following were blank, except for the name of the day. The participants were asked to describe situations and experiences in their work that they believed to contain some form of “ethical consideration”. We also asked for ideas and thoughts inspired by these considerations. The instructions deliberately contained no definition of either “ethics” or “considerations” as the idea was to enable the participants to feel free in relation to these concepts, in order to obtain spontaneous, freely provided statements. We suggested that they should write down their experiences every day after finishing work.

### Analysis and interpretation

Qualitative theory-guided content analysis was used in order to capture a description of staff encounters with patients and in what way these encounters were consistent with, or contrary to, the three perspectives [20,21]. 173 persons handed in their diaries containing information about 540 days of work. The participants in adult psychiatry (105 persons) were mental health care assistants (50%), registered nurses (39%) and other professionals, for instance psychiatrists, psychologists and social workers (11%), 85% had worked in psychiatric care for more than 3 years, their average age was 44 years and 74% were women.

Engstrom [4] previously conducted a more extensive review of these three perspectives. NVivo8 was used to identify all the values in this review. Based on this analysis we obtained an initial list of 28 values (Table 2), which were used as initial codes [20]. This was the theoretical base used for the empirical analysis. All statements in the diaries that contained any kind of description of encounters with patients were analysed, however, statements that had no reference to a patient were omitted. NVivo 8 was used to categorize the statements that then were placed in one of the three perspectives on the basis of the balance of power that was described. They were finally placed in a code that the statement referred to. To obtain a manageable amount of codes we merged codes that had many statements in common and that seemed to relate to similar value issues. The final codes were labelled in a way that yielded a “thick description” of the value/s they represented [22] (Table 3).

**Table 2:** 15 of the 28 initial values in the code list prior to the analysis of the empirical data

**Perspectives**	**Initial codes**
Paternalism	In the best interest of the patient
	Promoting and restoring health
	Providing relief and comfort
	Delivering good care
	Professional competence and integrity
Autonomy	Informing the patient
	Accepting the decisions of competent patients
	Respecting autonomy
	Respecting integrity
	Protecting human rights
Reciprocity	Participation
	Confidence
	Cooperation
	Consensus
	Involvement

**Table 3:** An example of the coding of a statement

**Perspective**	**Initial code**	**Statement**	**Final code**
	**/value**		**value**
Paternalism	Professional competence and integrity	At work tonight a patient was catastrophising about all the terrible things that happened to her relatives + agitated and verbally threatening towards staff. It’s difficult to approach her without offending her. How should I encounter the patient in her suffering?	To take responsibility

### Ethics

With the ethical diary followed instructions and a letter with information about the background and purpose of the study, and that participation was voluntary. The adult study was approved by the Regional Ethical Review Board in Uppsala (dnr 2008/017). The child and adolescent study was reviewed, according to previous regulations for research ethics review, by all of Sweden’s research ethics committees for medical research and was approved as a multi-center study by the committee in Orebro (reg. 411–02).

## Results

In our data we found that the majority of the statements were examples of the perspective of paternalism, while several also referred to autonomy. However, only a few could be attributed to reciprocity. The result is related to the three perspectives and contains eight values (Table 4).

•*Paternalism;* 1) promoting and restoring the health of the patient, 2) providing good care and 3) assuming responsibility.

•*Autonomy;* 1) respecting the patient’s right to self-determination and information, 2) respecting the patient’s integrity and 3) protecting human rights.

•*Reciprocity;* 1) involving patients in the planning and implementation of their care and 2) building trust between staff and patients.

**Table 4:** The result comprising three perspectives and eight values

**Perspectives**	**Values**
Paternalism	Promoting and restoring the health of the patient
	Providing good care
	Assuming responsibility
Autonomy	Respecting the patient’s right to self-determination and information
	Respecting the patient’s integrity
	Protecting human rights
Reciprocity	Involving patients in the planning and implementation of their care
	Building trust between staff members and patients

Each value is illustrated by a quotation and the final quotation in every perspective is a reflection on working contrary to the value.

### Paternalism

The participants expressed the importance of working in the best interest of patients, which is fundamental in the perspective of paternalism. They wanted *to promote and restore the health of the patient.* If necessary, coercive treatment could be administered in the best interest of the patient. Legally approved coercion was rarely considered ethically problematic. However, informal coercion, such as psychological pressure involving persuasion or the threat of coercion more often gave rise to ethical considerations. One participant stated that she/he would want to receive medication by means of coercive measures if she/he was as ill as the patients on whom coercion was used.

The patient is very upset about being medicated, says that I’m lying about the way he feels when not on medication. His description of how unwell he feels when he has to take medication and of how well he copes without it is very convincing. Since the patient is in compulsory care, we can determine what is good and bad treatment. In this case it’s the exact opposite to how the patient experiences it.

The participants aimed to *provide good care*. They could take time to talk to and calm patients when required. Sometimes they lacked the time and the patient received medication instead. There were also strategies in place to prevent violence on the ward. They worried about patients who, in their opinion, did not have adequate support from the social services.

Meeting about a home placement for a patient on long-term leave. The municipality wants to terminate the patient’s present placement, while we consider the placement necessary on the basis of the forensic psychiatric compulsory care act. However, it is the municipality that pays and the officials want a different type of accommodation.

Some statements indicated that, at times, loyalty towards colleagues was more important than the patient’s care and safety. However, a few statements revealed that some staff members were loyal to the patient rather than the organisation, despite being aware of the fact that their actions could lead to problems with colleagues.

The participants also wanted to *assume responsibility* for the patients and their well-being. Some went beyond their professional role by taking more responsibility for patients than was expected. When patients were unable to assume responsibility for their actions, the staff could take over. Sometimes this could lead to situations where patients in voluntary care were treated as if they were in coercive care. Responsibility could also mean that staff members had to take and implement difficult decisions against a patient’s will, including coercive measures that could be emotionally very unpleasant. Many of these paternalistic statements convey an impression of staff members acting in a way that they believe is beneficial for the patient. However, taking over the patient’s responsibility sometimes resulted in a punitive attitude that was found in some statements.

Last week a patient hit a staff member on the head with a plastic bottle. The patient’s behaviour is entirely inappropriate and inexcusable and his permission to go on leave was withdrawn. // Is this a punishment or an educational strategy. In my view it is both, but above all the purpose is to emphasise that such behaviour is unacceptable.

Many participants seemed to be aware of the risk of developing such an attitude, as in their diaries they were critical of colleagues who acted in this way.

### Autonomy

The participants emphasized the need to *respect the patient’s right to self-determination and information*. Several threats to patient autonomy were reported. One was lack of resources, which sometimes resulted in limitations on patient autonomy. Being permitted to go outdoors and other activities could be limited if staff members lacked the time to assist. Another risk was that ward practices, routines and rules were often extensive, and patients and staff were expected to respect them. Relatives could request information about the patient, which was problematic if the patient did not wish to inform them. Occasionally the staff decided to withhold information from patients, for example incoming mail or details about their care planning, in order not to create anxiety on the ward. Ward routines included conducting diagnostic tests, and at times management demanded tests, but it could happen that patients refused to participate.

Should we persuade a patient to undergo a test to see if she/he has a diagnosable disease when she/he doesn’t want to? This is a difficult question, which often arises. Should the patient be allowed to continue to live with the belief that she/he is functioning like the rest of us or made to undergo tests and probably receive a diagnosis, leading to an entitlement to more support and help?

The participants emphasised the importance of *respecting the patient’s integrity*, but also described ward routines that violated patient integrity. They reflected on how to perform certain tasks in a way that minimises violation of integrity, e.g. when carrying out a body or strip search as well as continuous observation.

A young female patient under continuous observation expresses that it is a violation not to be allowed to close the toilet door. Compromise; a 5 cm gap so that I can see her knees and she is told to turn on the tap in the washbasin, which she does and thinks it helps somewhat.

There were also statements revealing thoughts or behaviours where the participant or a colleague had no or only little respect for patient rights. Some participants perceived violations by colleagues and criticized their behaviour or language. Nevertheless, none of the participants actually noted down that they had reported violations against patients to their superiors and only a few considered doing so.

Some statements pertained explicitly to *protecting human rights.* There was criticism of the slow handling of asylum cases in Sweden and lack of respect for the patient’s right to her/his own opinions and religious beliefs.

The other day I had a discussion with a colleague about accompanying a patient who is a Muslim to the mosque. But he stated emphatically “I won’t do that because I don’t believe in it”. Sounds a little strange to my ears because it’s not about participating in a religion you don’t believe in, it’s about support and encouraging activity. Freedom of religion?

### Reciprocity

The participants described striving to *involve patients in the planning and implementation of their care*. One way to do this was to listen to the patients and ask about their wishes. Another was to let the patient choose her/his contact person. Although staff members felt pressure from management and colleagues to maintain a professional distance to patients, some participants, especially in child and adolescent psychiatry, chose to have a closer relationship with patients. However, they did not tell colleagues about their commitment to patients in order to avoid criticism.

I received an e-mail from the little Iranian girl today. I have a great relationship with her. My previous manager criticised me because I was “too involved”. That’s why I don’t always say how much contact I have with young people, even after they have been discharged from the clinic. // How formal should we be in psychiatry? It is difficult. Being spontaneously happy and friendly is not considered an asset in this “sick” world.

The participants expressed the need to *build trust between staff members and patients.* They could become frustrated when they failed to create a good relationship with patients and, in the case of child and adolescent psychiatry, with parents. Some participants were committed to their work and clearly shared the joy of patients when they were happy or the treatment outcome was positive.

Before breakfast; one of my “old” patients came to be weighed. Weight gain, great joy, both for me and the patient.

Some participants found it very difficult to control their negative feelings about certain seriously ill patients. They did not want any kind of reciprocity and instead sought to distance themselves from these patients. They used objectifying language about patients such as “one anorectic” or “a suicide candidate” instead of “patient”.

I’m the contact person for a guy who committed a crime. He has no empathy towards his victim. I have negative thoughts about him, but of course I don’t show anything. I find it very hard to cope with disturbed people.

## Discussion

The three concepts; paternalism, autonomy and reciprocity, reflect different ethical perspectives in the development of medical ethics in psychiatry. There is a long tradition of paternalism in psychiatry, but patient rights and reciprocity have been considerably more in focus in recent decades. Nevertheless, in this study, paternalism clearly appeared to be the dominant perspective in the diaries analysed. The participants were also aware of patients’ right to autonomy. However, it was difficult to find statements about reciprocity and, of the few identified, most were reflections on staff members working contrary to reciprocity. The reason for this overrepresentation of paternalism in the diaries might be that staff members are aware of the importance of autonomy. Therefore they consider it problematic to act in a paternalistic fashion, although in certain situations it seems to be the only appropriate way. Another reason could be the ongoing reduction in the number of psychiatric beds in Sweden [23]. Today, in-patients are in a worse condition than previously and the proportion cared for by coercion has increased. There are more patients who are incapable of taking care of themselves and making their own decisions. So, the need for a paternalistic perspective and a substitute decision-making may be more necessary today than previously, when patients were not as seriously ill and therefore more capable of assuming responsibility.

There were normative statements that revealed an awareness of patient rights and how staff should behave in order to respect them. However, when describing encounters with patients, the participants stated that living up to such ideals was difficult. In this study as in previous research [24], there were examples of staff members who more or less routinely failed to respect the right to autonomy, including that of patients in voluntary treatment. However*,* in the present study there were also many who criticized such behaviour as well as the use of objectifying language to refer to patients and others.

As argued earlier, the three perspectives should be seen as complementary, representing different valuable contributions. The third and most recent perspective, reciprocity, can make a useful contribution to psychiatry because it focuses on values that have been lacking in psychiatric practice. Both the normative literature [15] and psychiatric law in, e.g. Sweden and the UK [25,26], stress that reciprocity, even in coercive care, should be seen as a core value in psychiatric care and that staff should involve patients in all stages of care and treatment, also in coercive care. However, shifting from paternalism or autonomy to reciprocity seems to be difficult in practice, as it implies moving from a perspective where only one person, either the professional or the patient, is in focus, to a dyadic relation in which the focus is on the interaction between the professionals, the patient and her/his family. A way to foster reciprocity in psychiatry may be to consider it as value-based practice [15]. Two features of value-based practice are to 1) always start with the patient’s perspective but also seek a balance between legitimately different perspectives and 2) ensure that communication skills play a substantial role in clinical decision-making. In psychiatry it is not always possible to achieve reciprocity in decision-making. However*,* staff should at all times try to achieve an open dialogue to reach a compromise that is acceptable to the patient as well as adequate enough from a professional perspective. Only after such a strategy has failed should staff consider paternalistic decision-making [3]. Empirical research has demonstrated the great importance of the patient perceiving that staff members genuinely care about and listen to her/him [27,28]. The opportunity to participate makes the patient feel like a valued and normal human being, while lack of participation makes her/him feel of less value than other people. Studies have found [28,29] that patients who appreciated the commitment on the part of staff rarely perceived that they were subject to coercion in comparison with those who did not consider staff members to be committed. Thus the sense of being subject to coercion was not directly related to whether or not the care was voluntary. When carrying out coercive measures it is especially important to talk to the patient, not because it necessarily changes the situation but makes her/him feel respected as a human being [27,29].

Despite the normative trend towards reciprocity in the ethics of psychiatry throughout the Western world, it was difficult to find examples of reciprocity in this study, even though the participants were employed in thirteen independent clinics. In the present study, reciprocity seems to be something that only some staff members strive for, especially in child and adolescent psychiatry. Sometimes they felt that they had to keep it secret, due to the risk of being criticized by colleagues and superiors if they became too involved with patients. On the basis of this study, it is impossible to establish how frequently the principle of reciprocity is not respected. However, our result is worth bearing in mind when clinics are considering the need for ethical education, training or supervision. Ethical diaries could be useful for education and discussions with practitioners.

This study indicates that the three ethical perspectives were helpful when analysing the empirical data. As the diaries contain so many statements, they present a spectrum of ethical considerations in daily work in psychiatric in-patient care. However, there are some methodological limitations. Most of the statements are short, which makes it difficult to fully understand the underlying process. For instance, it was difficult to assess the presence of shared decision-making. The statements often highlighted a critical event but it was seldom possible to follow a longer process. Another limitation of this study is that it gives only the staff’s view of the encounters. There is also a need to conduct research on how patients perceive encounters with professionals in psychiatry. Future research in this area should use methods that allow the values in psychiatry to be examined in greater detail, as well as analyse how the three decision-models are used in practice.

## Conclusions

Paternalism appeared to be the dominant perspective among the participants, but there was also an awareness of patients’ right to autonomy. Despite the trend towards reciprocity in normative ethics of psychiatry throughout the Western world, identifying it in practice proved difficult*.* This should be borne in mind when clinics consider the need for ethical education, training and supervision.

## Competing interests

The authors declare that they have no competing interests.

## Authors’ contributions

IE, a child and adolescent psychiatrist, formulated the research idea, designed the study and was responsible for the data collection. VP, a social worker, conducted the analysis, all stages of which were scrutinized by KE, a pedagogue. Some amendments were made by consensus after discussion with all authors. VP drafted the manuscript. All authors have contributed to, read and approved the manuscript.

## Pre-publication history

The pre-publication history for this paper can be accessed here:

http://www.biomedcentral.com/1472-6939/14/49/prepub
